# The 25th Norwegian symposium on chromatography

**DOI:** 10.1002/ansa.202300035

**Published:** 2023-07-08

**Authors:** Helle Malerød‐Fjeld, Trine Grønhaug Halvorsen

**Affiliations:** ^1^ Department of Forensic Sciences Oslo University Hospital Oslo Norway; ^2^ Organizing committee Norwegian Chemical Society's Norwegian symposium on chromatography Bærum Norway; ^3^ Department of Pharmacy University of Oslo Oslo Norway

From September 4 to 6, 2022, the 25th Norwegian symposium on chromatography took place in Sandefjord, Norway. The meeting series is organized every second year under the auspices of the Norwegian Chemical Society, Department of Analytical Chemistry.

The biannual Norwegian symposium on chromatography is a key meeting for the Norwegian analytical chemistry environment. The series of chromatography symposia in Norway started in 1974 with a meeting in gas chromatography, and in 1980, the symposium changed name to the Norwegian symposium on chromatography to include all chromatographic techniques. Since then, meetings have been held regularly, biannually from 1982, in Sandefjord, Norway.

The Norwegian symposium on chromatography is known for a high scientific level, and for pleasant social activities including a symposium dinner and nightclub activities. With its mix of social and scientific program, it is a popular meeting place for the entire Norwegian chromatographic environment, gathering around 200 participants every time.

The 25th jubilee symposium was first postponed from January to September due to Covid‐19 and, hence meeting restrictions. Luckily when September approached the Covid‐19 restrictions were loosened and the symposium could be held as planned without restrictions in numbers and need for keeping distance.

The 25th symposium was chaired by Karina Langseth‐Manrique, with Helle Malerød‐Fjeld and Trine Grønhaug Halvorsen as co‐chairs responsible for the scientific program. In addition, Espen Storbråten and Sonny Langseth were part of the organizing committee. Almost 200 participants working in industry, hospitals and academia attended the symposium. Over the course of the 3 days of the symposium, 41 oral presentations were presented. These covered fundamental and practical aspects of chromatographic techniques, including high performance liquid chromatography (HPLC), gas chromatography (GC), liquid chromatography mass spectrometry (LC‐MS), supercritical fluid chromatography (SFC), a variety of sampling and sample preparation techniques, and applications in food analysis, environmental analysis, bioanalysis and doping analysis. In addition to the lectures, the participants could attend 12 elevator pitches from vendors, four short‐courses in basic HPLC, basic LC‐MS, practical maintenance and troubleshooting in gas chromatography, and data integrity, as well as a poster section. The symposium is also important for the vendors (and vice versa), and this year the exhibition counted 22 vendors in the exhibition hall. Here are some of the highlights of the symposium:

1. Excellent plenary talks by five well renowned international and four national speakers.
2. Dedicated parallel sessions to young emerging scientists, with many excellent talks and poster session with great contributions from Master's students, PhD students and other researchers.
3. Social activities including sparkling wine tasting and concert with *Echoes*, a band with members from the Norwegian chromatography community playing music from among others Pink Floyd, The Beatles, Rolling Stones.
4. The award “The Golden Column” was awarded to Cato Brede (Stavanger University Hospital) for his contribution to the symposium during the past 25 years

The symposium started with an opening plenary lecture from Deirdre Cabooter (Catholic University of Leuven, Belgium) focusing on machine learning (e.g., deep learning) techniques to automate the different steps required to develop new LC methods. Her lecture was followed by a talk by Thomas Gundersen, CEO and co‐founder of Vitas Analytical Services, Norway talking about his and his company's journey from a small start‐up 30 years ago until being a recognized international contract research lab today. The other invited international speakers were Sebastiaan Eeltink (Free University of Brussels, Belgium), Charlotta Turner (Lund University, Sweden), Margrét Þorsteinsdóttir (University of Iceland, Iceland) and Jan H. Christensen (University of Copenhagen, Denmark) all giving excellent talks.

During the symposium dinner awards were given for the three best posters and the best oral presentation by a young scientist (the young scientist award). The latter award was co‐sponsored by Analytical Science Advances. The winners of the poster awards were Christina Johannsen (University of Oslo) with her poster describing paper‐based immunocapture in targeted LC‐MS‐based protein biomarker analysis, Alexander Bauer Westbye (Oslo University Hospital) presenting a method for determination of free thyroid hormones in serum by equilibrium dialysis LC‐MS/MS and Sander Guttorm and Cristina Alexandrescu (University of Oslo) presenting how to evaluate the metabolome concentration stability on dried blood spot (DBS) cards by nuclear magnetic resonance (NMR) and LC‐MS. The winner of the Young scientist award Christine Olsen (University of Oslo) presented her PhD research about online determination of insulin response in human pancreatic islet. An interview with Christine can be found in a separate editorial. In addition, travel and participation to the 51st International Symposium on High Performance Liquid Phase Separations and Related Techniques (HPLC 2023) in Dusseldorf, Germany was drawn between the participants at the symposium. The lucky winner of travel and participation to HPLC2023 was May Helene Engebretsen, Senior QC Advisor at Takeda, Norway. Glimpses from the symposium can be found in Figure 1. We are now already looking forward to the next Norwegian symposium on chromatography January 21 to 23, 2024.

**FIGURE 1:**
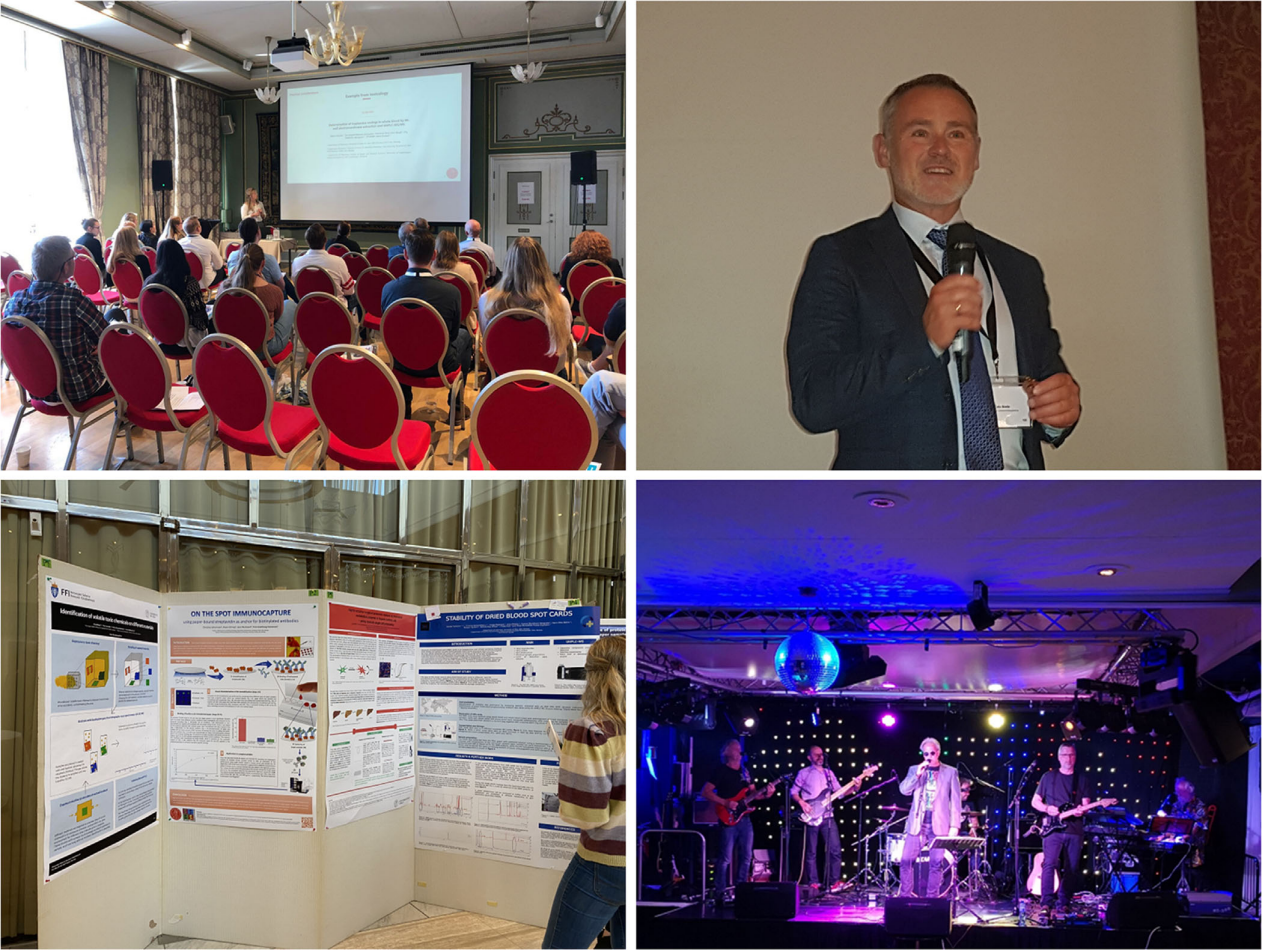
Glimpses from the symposium: Lectures (top left); Cato Brede, the recipient of «The Golden Column» (top right); poster session with the poster receiving the 1st place for best poster as number two from left (bottom left); concert by *Echoes* (bottom right).

In this special issue devoted to the Norwegian symposium on chromatography, you will find contributions describing research presented at the symposium. This is in addition to the interview with Christine Olsen, recipient of the young scientist award. A common theme of all the contributions is bioanalysis, and especially sampling and sample preparation. Skogvold et al. are in their research article describing how preanalytical factors such as storage media, for example whole blood versus dried blood spots as well as kind of blood spot paper can affect the results in global metabolomics studies. This is of high importance in studies involving rare diseases or medical conditions as the inclusion time often is long and the sampling and storage conditions can vary. Sampling and sample preparation is also the theme of Reubsaet and Halvorsen's perspective. They present and discuss the smart sampling approach for more efficient liquid chromatography mass spectrometry‐based bioanalysis of proteins. Using this approach common sample preparation steps for protein analysis such as proteolysis and affinity clean‐up are integrated with the sampling. This frees time and labor after arrival of the samples in the analytical laboratory. In the tutorial by Schüller et al. the reader will get fundamental understanding of the green sample preparation technique electromembrane extraction. In electromembrane extraction an electric field is applied to extract charged analytes from an aqueous sample through a liquid membrane and into an aqueous acceptor. The turorial provides the reader with the tools needed for method development and operation, and how to avoid common pitfalls. The aim of Westbye et al.’s mini‐review is to make implementation of liquid chromatography tandem mass spectrometry methods for determination of free thyroid hormones in the clinical laboratory more easy. The medical rationale for free thyroid hormone determination is given as well as the benefits of liquid chromatography tandem mass spectrometry compared to immunoassay‐based methods. Important parameters to obtain physiologically relevant free thyroid hormone concentrations are also highlighted.

## CONFLICT OF INTEREST STATEMENT

The authors declare that there is no conflict of interest that could be perceived as prejudicing the impartiality of the research reported.

## Data Availability

Data sharing not applicable ‐ no new data generated.

